# Evaluating the Performance of ChatGPT-4o Oncology Expert in Comparison to Standard Medical Oncology Knowledge: A Focus on Treatment-Related Clinical Questions

**DOI:** 10.7759/cureus.78076

**Published:** 2025-01-27

**Authors:** Oguzcan Kinikoglu, Deniz Isik

**Affiliations:** 1 Medical Oncology, Kartal Dr. Lütfi Kirdar City Hospital, Health Science University, Istanbul, TUR

**Keywords:** artificial intelligence and education, chatgpt-4o, clinical-decision making, oncology, oncology expert

## Abstract

Integrating artificial intelligence (AI) into oncology can revolutionize decision-making by providing accurate information. This study evaluates the performance of ChatGPT-4o (OpenAI, San Francisco, CA) Oncology Expert, in addressing open-ended clinical oncology questions. Thirty-seven treatment-related questions on solid organ tumors were selected from a hematology-oncology textbook. Responses from ChatGPT-4o Oncology Expert and the textbook were anonymized and independently evaluated by two medical oncologists using a structured scoring system focused on accuracy and clinical justification. Statistical analysis, including paired t-tests, was conducted to compare scores, and interrater reliability was assessed using Cohen’s Kappa. Oncology Expert achieved a significantly higher average score of 7.83 compared to the textbook’s 7.0 (p < 0.01). In 10 cases, Oncology Expert provided more accurate and updated answers, demonstrating its ability to integrate recent medical knowledge. In 26 cases, both sources provided equally relevant answers, but the Oncology Expert’s responses were clearer and easier to understand. Cohen’s Kappa indicated almost perfect agreement (κ = 0.93). Both sources included outdated information for bladder cancer treatment, underscoring the need for regular updates. ChatGPT-4o Oncology Expert shows significant potential as a clinical tool in oncology by offering precise, up-to-date, and user-friendly responses. It could transform oncology practice by enhancing decision-making efficiency, improving educational tools, and serving as a reliable adjunct to clinical workflows. However, its integration requires regular updates, expert validation, and a collaborative approach to ensure reliability and relevance in the rapidly evolving field of oncology.

## Introduction

Integrating artificial intelligence (AI) in healthcare, particularly in medical oncology, has sparked significant interest and debate. Among the AI tools gaining attention is ChatGPT (OpenAI, San Francisco, CA), which has shown potential in various medical applications. Its unique ability to generate human-like text based on prompts has led to its exploration in diverse areas of healthcare, ranging from clinical decision-making to patient education. This article evaluates a specialized AI called “Oncology Expert,” accessed through ChatGPT 4, to explore GPT's approach to addressing medical oncology problems, comparing its capabilities with those of experienced medical oncologists.

Recent studies have explored the effectiveness of ChatGPT in the telemedicine-based management of metastatic prostate carcinoma, revealing a moderate concordance with oncologists in treatment decisions [1,2]. ChatGPT's ability to pre-chart and determine the necessity of face-to-face consultations has been assessed, showing potential time savings and moderate agreement in clinical decision-making [1]. Additionally, ChatGPT has been evaluated as a clinical resource for medical oncologists, demonstrating partial success in answering board-style questions and real-world patient cases. However, it fell short of the non-inferiority margin set for these evaluations [3]. These findings illustrate the strengths and limitations of AI applications in dynamic and high-stakes clinical environments where precision and reliability are critical.

The application of AI in oncology is further highlighted by studies examining ChatGPT's performance in clinical decision-making and its concordance with oncologists' treatment decisions. These studies underscore the challenges and variability in AI-generated recommendations, emphasizing the need for further research to enhance AI's reliability and applicability in personalized patient care [4,5]. Furthermore, using AI tools has raised ethical considerations, such as ensuring transparency in decision-making processes, mitigating biases in AI-generated outputs, and maintaining patient trust in AI-assisted care [6]. Addressing these issues is essential to safely and effectively integrating AI into clinical workflows.

Moreover, specialized models like OncoGPT, trained on American Society of Clinical Oncology (ASCO) guidelines, have been developed to provide precise and guideline-adherent oncological advice, showcasing the potential for AI to support oncology professionals with accurate and contextually relevant information [7]. Such tailored models offer a glimpse into how domain-specific AI systems could further narrow the gap between human expertise and machine-assisted insights, highlighting opportunities for improving efficiency and accuracy in clinical settings.

Despite the promising capabilities of ChatGPT and similar AI models, limitations remain, particularly in generating personalized treatment plans and maintaining consistency with human physicians' recommendations [8]. AI in oncology does not replace medical professionals but augments their capabilities, offering support in managing complex patient cases and improving the overall patient experience [9]. As the field evolves, it will be imperative to establish robust training datasets, develop sophisticated algorithms, and ensure ongoing validation to maximize the utility of AI in oncology.

In conclusion, while ChatGPT and other AI models present intriguing possibilities for enhancing oncological decision-making, their integration into clinical practice requires careful consideration of their limitations and ongoing validation through rigorous research. This study specifically addresses the gap in understanding whether ChatGPT-4o Oncology Expert can provide responses to open-ended (OE) clinical oncology questions that are not only accurate and evidence-based but also clear and up to date. By directly comparing its performance to a standard oncology textbook, the study aims to assess the model’s potential as a practical tool for supporting oncology education and clinical decision-making.

## Materials and methods

Study design

This study was designed to compare ChatGPT-4’s “Oncology Expert” performance against established textbook medical oncology knowledge. OE treatment-related questions were selected from the Hematology-Oncology Clinical Questions textbook (McGraw Hill Press, New York, NY) [10]. Only questions related to solid organ tumors were included. A total of 37 treatment-related clinical questions were chosen without modification. Responses were generated from ChatGPT-4o Oncology Expert, which was accessed through the Explore GPT platform using its oncology-specific prompt mode, and book-derived standard answers [11]. Each question was entered verbatim without rephrasing, and no preprocessing was performed. AI responses were captured as-is, with minor formatting adjustments (e.g., removing extraneous text) for clarity, but the content remained unaltered.

These answers were randomized and anonymized to prevent bias and subsequently presented to two evaluators: an experienced internal medicine specialist and medical oncologist (M.D D.I.) with nine years of specialization in medical oncology and a senior medical oncology fellow and internal medicine specialist (M.D O.K.) with five years of experience in internal medicine and a senior resident in medical oncology. Each response was evaluated independently. To address potential bias arising from O.K.'s prior exposure to both sources, the randomization and anonymization processes ensured that neither evaluator could identify whether an answer originated from the AI or the textbook. Additionally, O.K. strictly adhered to the structured scoring system during the evaluation and refrained from revisiting the original sources while scoring. Both evaluators were allowed access to current clinical guidelines during the evaluation process to minimize bias and ensure fair comparisons. Minor omissions were defined as missing details that did not alter clinical recommendations, whereas “significant gaps” referred to errors or missing information that could lead to inappropriate clinical decisions. When disagreements arose, evaluators discussed the scores, referring to guidelines or textbooks, and reached a consensus through joint review. To ensure objective comparison, a structured scoring system was developed based on the following criteria.

Accuracy (0-4 points)

4: Completely accurate and adheres to current guidelines and standards of care.

3: Mostly accurate with minor omissions or discrepancies.

2: Partially accurate; includes relevant points but significant gaps.

1: Inaccurate, providing incorrect or outdated information.

0: No relevant answer or completely incorrect response.

Clinical Justification (0-4 points)

4: Well-reasoned explanation with evidence-based rationale.

3: Adequate justification with minimal omissions.

2: Partial explanation lacking clarity or depth.

1: Limited or unclear justification.

0: No rationale provided.

In cases where discrepancies in scoring occurred, a resolution protocol was implemented: the evaluators reviewed the question together, referring to the most recent clinical guidelines to reach a consensus. This approach ensured that scoring differences were resolved systematically and that the final scores reflected the most accurate and contextually appropriate responses.

Development and training of ChatGPT-4o Oncology Expert

The ChatGPT-4o Oncology Expert is a domain-specific iteration of OpenAI’s GPT-4 architecture, fine-tuned on a corpus of oncology-related literature, including peer-reviewed journals, clinical guidelines (such as National Comprehensive Cancer Network (NCCN), ASCO, and European Society for Medical Oncology (ESMO)), and textbooks. The model underwent supervised learning from these resources, supplemented by reinforcement learning from human feedback (RLHF). This approach aimed to enhance its ability to provide accurate, contextually relevant, and guideline-concordant responses to clinical queries.

The model incorporates contextual reasoning to align its recommendations with established oncology practices, but it remains dependent on the breadth and currency of its training data. The training cut-off, reflective of publications available as of 2024, highlights the need for ongoing updates to maintain alignment with emerging evidence and evolving guidelines.

Limitations of ChatGPT-4o Oncology Expert

While the model demonstrated superior performance in oncology, its inherent limitations must be acknowledged. First, the static nature of its training data makes it susceptible to outdated recommendations. Additionally, the model lacks access to real-time clinical information, such as imaging or pathology results, which limits its applicability in comprehensive diagnostic decision-making. Lastly, ChatGPT-4o's reliance on probabilistic outputs means its responses, while often accurate, may occasionally lack depth or context specific to nuanced clinical scenarios.

Statistical analysis

To analyze the data, descriptive statistics were employed to summarize the overall performance of ChatGPT-4o Oncology Expert, including means, standard deviations, and concordance rates. Paired t-tests were conducted to compare the performance scores of ChatGPT-4o Oncology Expert and the book-derived answers. Inter-rater reliability was calculated using Cohen’s Kappa (κ) to assess the consistency in scoring. Statistical significance was set at p<0.05 for all analyses, which were carried out using Python (version 3.13.0; Python Software Foundation, Wilmington, DE).

Before performing paired t-tests, the normality of the score distributions was assessed using the Shapiro-Wilk test. No significant outliers were identified, and the assumptions for the paired t-test were met. Cohen’s Kappa was calculated to assess inter-rater reliability, and its interpretation followed standard thresholds.

The study adhered to ethical guidelines, including anonymizing responses to protect confidentiality. No ethical approval was required for this study, as it did not involve patient data or clinical trials.

## Results

In this comparative evaluation of ChatGPT-4o Oncology Expert’s performance against the established medical oncology knowledge from the Hematology-Oncology Clinical Questions textbook (McGraw Hill Press), 37 treatment-related OE clinical questions were assessed. Both ChatGPT-4o and the book-derived answers were independently evaluated by medical oncologists using a structured scoring system based on accuracy and clinical justification with the book answer.

The textbook answers achieved an average total score of 7.0 points per question, whereas ChatGPT-4o Oncology Expert outperformed with a higher average score of 7.83, as evaluated by the reviewers. This difference highlights ChatGPT-4o Oncology Expert’s ability to offer more accurate and updated answers in most cases. A paired t-test statistical analysis confirmed that ChatGPT-4o Oncology Expert’s performance was significantly better, with a p-value <0.01, indicating a strong difference between the two sources. 

For both evaluators in 10 of the 37 clinical cases, ChatGPT-4o Oncology Expert provided more accurate and updated answers compared to the textbook, showing its capacity to integrate more recent medical knowledge effectively. The evaluators disagreed in only one case: D.I. rated ChatGPT-4o Oncology Expert superior, while O.K. assessed it as equivalent to the textbook answers. In 26 cases, both sources provided equally relevant and clinically appropriate responses. However, even in these instances, ChatGPT-4o Oncology Expert outperformed the textbook in terms of clarity and ease of understanding. This aspect of clarity is particularly important for educational purposes, as it enhances the ability of learners to grasp complex medical concepts.

Inter-rater reliability was assessed using Cohen’s Kappa, which showed a perfect agreement (κ = 0.93) between the evaluators' ratings on questions about whether ChatGPT-4o Oncology Expert was “better” than the textbook. This high level of consistency underscores the reliability of the evaluation process and strengthens the validity of the results.

Notably, in the case concerning metastatic bladder cancer treatment, both ChatGPT-4o Oncology Expert and the textbook provided outdated information. Despite this, the book’s response was not considered superior (Table 1).

**Table 1:** Summary of ChatGPT-4o Oncology Expert vs textbook evaluation results

Criteria	Results
Number of questions evaluated	37
Average score (textbook)	7.0
Average score (ChatGPT-4o Oncology Expert)	7.83
Cohen’s kappa (inter-rater reliability)	0.93
Cases where ChatGPT-4o was superior	10
Cases where both were equivalent	26
Cases with disagreement between evaluators	1

## Discussion

This study highlights the potential of ChatGPT-4o Oncology Expert as a valuable tool in oncology, particularly accurate and updated answers to OE clinical life questions that demand nuanced reasoning rather than reliance on pre-structured multiple-choice (MC) formats. In 10 out of the 37 clinical cases, ChatGPT-4o Oncology Expert provided more accurate and updated answers than the textbook, while maintaining an impressive overall accuracy of 97.3% by correctly answering 36 of 37 cases. This underscores its ability to integrate up-to-date medical knowledge effectively and reflects its adaptability to the rapidly evolving oncology landscape. In contrast, the textbook, despite its reputable standing, lagged in providing the most current treatment information, underscoring the challenge of ensuring textbooks remain up to date with evolving clinical guidelines. The higher average score of ChatGPT-4o Oncology Expert (7.83 points) over the textbook’s answers (7.0 points) suggests that AI models can offer more precise and contextually relevant responses in complex clinical scenarios. This is consistent with previous research, indicating that AI models can perform well in medical examinations and real-world patient cases, showcasing their potential as clinical resources [3,12]. For instance, ChatGPT demonstrated a 62% accuracy rate in answering MC questions and a 43.8% accuracy rate in OE questions from the ASCO Self-Evaluation Program. Notably, its performance varied significantly across specialties, achieving its highest accuracy in malignant hematology (81.8% for MC, 72.8% for OE) but struggling with genitourinary malignancies (60% for MC, 20% for OE). Additionally, when addressing real-world patient case questions, ChatGPT’s responses aligned with clinicians in 50% of cases, with 80% of those concordant responses providing sufficient detail for clinical decision-making [7]. Complementing this, GPT-4 achieved an impressive 84.4% accuracy on ASCO and 86.7% on ESMO trial questions, significantly outperforming GPT-3.5 (57.8% for ASCO and 65.3% for ESMO), further demonstrating its potential as a reliable resource in oncology education and practice [12].

Studies further highlight that GPT-4 surpasses earlier versions and other language models, achieving a 20% higher margin on the USMLE and scoring in the 90th percentile for the SAT, GRE verbal section, and Uniform Bar Exam, showcasing its advanced reasoning and contextual understanding capabilities​ [13]. AI tools like ChatGPT-o1 and Gemini 2.0 Advanced (Google DeepMind, London) demonstrated exceptional accuracy on the Turkish Dental Specialty Examination, achieving 97.46% and 97.90% accuracy, respectively, significantly outperforming ChatGPT-4o, which scored 88.66% (p = 0.0016), and Gemini 1.5 Pro, which scored 91.60% (p = 0.0007). ChatGPT-o1 and Gemini 2.0 also surpassed the highest human scores in both years [14]. 

The superior performance of ChatGPT-4o Oncology Expert in this study, achieving a remarkable 97.3% accuracy in clinical cases, compared to the 84.4% and 86.7% scores on ASCO and ESMO trial questions by GPT-4, illustrates the potential benefits of domain-specific training and tailored optimization. The specialized fine-tuning of ChatGPT-4o for oncology-specific contexts likely played a pivotal role in its enhanced accuracy and relevance.

In 26 of the 37 cases, both ChatGPT-4o Oncology Expert and the textbook provided equally clinically relevant responses. However, even in these cases, ChatGPT-4o Oncology Expert offered more comprehensible and easier-to-understand answers compared to the textbook. Although this study was not specifically designed to evaluate the clarity and educational value of the responses, it is worth noting that, as both educators and learners, we should consider the importance of these aspects in clinical education and decision support. This is an important aspect, especially for educational purposes, as it highlights the potential of AI models to present complex medical information in a more accessible and user-friendly manner. Although AI can outperform traditional resources in many instances, it still requires oversight to ensure the accuracy and applicability of its recommendations. The need for expert-driven verification is a recurring theme in the literature, emphasizing the role of AI as an adjunct rather than a replacement for human expertise [15,16].

A particularly important finding was the outdated information provided in the bladder cancer treatment case by both ChatGPT-4o Oncology Expert and the textbook. In this instance, neither source reflected the most recent advancements in treatment protocols, highlighting a critical area for improvement. For example, while newer guidelines recommend enfortumab vedotin and pembrolizumab as first-line therapy from the beginning of 2024 for metastatic bladder cancer, both sources relied on older treatment regimens. But still, ChatGPT-4o Oncology expert presented outdated information, which was still a more up-to-date treatment regimen than the textbook recommendation. This underscores the need for regular updates to AI systems and traditional resources to ensure alignment with evolving clinical evidence. This highlights the need for continuous updates and expert validation in AI-generated content [17,18]. 

A key observation in this study was the occurrence of outdated information in responses from ChatGPT-4o Oncology Expert and the textbook, particularly in the case of metastatic bladder cancer treatment.

Such examples emphasize the dynamic nature of oncology, where treatment paradigms rapidly evolve due to emerging clinical trial data. For AI systems like ChatGPT-4o Oncology Expert, this points to the importance of incorporating mechanisms for continuous learning and updating. Similarly, for textbooks, this highlights the inherent limitation of static content in keeping pace with real-time advances. Addressing these gaps is crucial to improving the reliability of clinical decision-making tools and resources.

The literature suggests that while AI models like ChatGPT-4o have shown promise, they still require improvements in handling complex clinical decision-making and ensuring the accuracy of their outputs [19,20]. 

Despite the promising results, this study has several limitations that warrant consideration. First, while ChatGPT-4o Oncology Expert demonstrated remarkable accuracy, its performance may not fully generalize to all clinical scenarios, as the study used only 37 clinical examples. A larger and more diverse dataset encompassing a broader spectrum of oncological conditions and complexities would provide a more robust evaluation of its capabilities. The use of OE questions in this study is essential for evaluating ChatGPT-4o Oncology Expert’s ability to generate nuanced, evidence-based responses. This approach mirrors real-world clinical scenarios, requiring the AI to synthesize information, reason contextually, and align with current guidelines, unlike MC formats. It also highlights the model's educational value in clarifying complex concepts and supporting medical professionals.

However, the study's scope is limited by the small set of 37 clinical questions, which reflects only a narrow subset of oncology topics. Additionally, the absence of multimodal data, such as imaging or genomics, restricts the evaluation of the AI's full potential in clinical practice. Expanding the range of questions and incorporating multimodal data in future studies will provide a more comprehensive assessment of its capabilities.

To evaluate the AI's ability to handle multimodal data, future studies could integrate imaging data (e.g., CT scans, MRIs) and genomic information (e.g., next-generation sequencing reports) alongside text-based clinical scenarios. This would require training AI models on combined datasets that include textual, visual, and molecular data. Developing hybrid evaluation frameworks that incorporate real-world oncology cases with multimodal inputs could offer a more comprehensive understanding of AI's utility in clinical practice.

Additionally, longitudinal studies should assess how AI tools like ChatGPT-4o influence clinical decision-making over time, especially in environments where medical professionals use them as adjuncts. Exploring patient outcomes, user satisfaction, and efficiency metrics could provide valuable insights into their real-world impact.

## Conclusions

This study demonstrates the potential of ChatGPT-4o Oncology Expert to provide accurate, user-friendly, and up-to-date responses to clinical oncology questions, highlighting its promise as an adjunct tool for oncologists. To facilitate the integration of AI tools like ChatGPT-4o into clinical workflows, several steps should be considered. First, establishing robust mechanisms for regular updates is essential to ensure that AI-generated content aligns with the latest clinical guidelines and evidence. This could involve integrating AI systems with dynamic databases that continuously update based on newly published studies and evolving treatment standards. Second, a collaborative approach should be adopted, where AI tools serve as decision-support systems rather than replacements for human expertise. Clear protocols for verifying AI recommendations, particularly in complex or high-stakes cases, will be crucial to maintaining accuracy and trust. Training programs for clinicians on how to effectively use AI tools in practice could further enhance their utility. Third, ongoing validation studies are needed to evaluate AI performance in real-world settings, focusing on key metrics such as impact on clinical decision-making, efficiency, and patient outcomes. Special attention should be given to how AI systems handle diverse and multimodal data inputs, ensuring their applicability to comprehensive oncology care.

By addressing these considerations, AI tools like ChatGPT-4o Oncology Expert could significantly enhance decision-making, streamline workflows, and improve the accessibility of evidence-based oncology care, provided their deployment is carefully managed and continuously validated.
